# Dual-modal radiomics nomogram based on contrast-enhanced ultrasound to improve differential diagnostic accuracy and reduce unnecessary biopsy rate in ACR TI-RADS 4–5 thyroid nodules

**DOI:** 10.1186/s40644-024-00661-3

**Published:** 2024-01-23

**Authors:** Jia-Yu Ren, Wen-Zhi Lv, Liang Wang, Wei Zhang, Ying-Ying Ma, Yong-Zhen Huang, Yue-Xiang Peng, Jian-Jun Lin, Xin-Wu Cui

**Affiliations:** 1 Department of Medical Ultrasound, Tongji Hospital, Tongji Medical College, Huazhong University of Science and Technology, Wuhan, China; 2 Department of Artificial Intelligence, Julei Technology Company, Wuhan, China; 3 Center of Computer, Tongji Hospital, Tongji Medical College, Huazhong University of Science and Technology, Wuhan, China; 4 Department of Medical Ultrasound, The First People’s Hospital of Qinzhou, Qinzhou, China; 5 Department of Medical Ultrasound, Wuhan Third Hospital, Tongren Hospital of Wuhan University, Wuhan, China

**Keywords:** ACR TI-RADS 4–5, Thyroid nodules, Contrast-enhanced ultrasound, Radiomics, Nomogram

## Abstract

**Background:**

American College of Radiology (ACR) Thyroid Imaging Reporting and Data System (TI-RADS, TR) 4 and 5 thyroid nodules (TNs) demonstrate much more complicated and overlapping risk characteristics than TR1-3 and have a rather wide range of malignancy possibilities (> 5%), which may cause overdiagnosis or misdiagnosis. This study was designed to establish and validate a dual-modal ultrasound (US) radiomics nomogram integrating B-mode ultrasound (BMUS) and contrast-enhanced ultrasound (CEUS) imaging to improve differential diagnostic accuracy and reduce unnecessary fine needle aspiration biopsy (FNAB) rates in TR 4–5 TNs.

**Methods:**

A retrospective dataset of 312 pathologically confirmed TR4-5 TNs from 269 patients was collected for our study. Data were randomly divided into a training dataset of 219 TNs and a validation dataset of 93 TNs. Radiomics characteristics were derived from the BMUS and CEUS images. After feature reduction, the BMUS and CEUS radiomics scores (Rad-score) were built. A multivariate logistic regression analysis was conducted incorporating both Rad-scores and clinical/US data, and a radiomics nomogram was subsequently developed. The performance of the radiomics nomogram was evaluated using calibration, discrimination, and clinical usefulness, and the unnecessary FNAB rate was also calculated.

**Results:**

BMUS Rad-score, CEUS Rad-score, age, shape, margin, and enhancement direction were significant independent predictors associated with malignant TR4-5 TNs. The radiomics nomogram involving the six variables exhibited excellent calibration and discrimination in the training and validation cohorts, with an AUC of 0.873 (95% CI, 0.821–0.925) and 0.851 (95% CI, 0.764–0.938), respectively. The marked improvements in the net reclassification index and integrated discriminatory improvement suggested that the BMUS and CEUS Rad-scores could be valuable indicators for distinguishing benign from malignant TR4-5 TNs. Decision curve analysis demonstrated that our developed radiomics nomogram was an instrumental tool for clinical decision-making. Using the radiomics nomogram, the unnecessary FNAB rate decreased from 35.3 to 14.5% in the training cohort and from 41.5 to 17.7% in the validation cohorts compared with ACR TI-RADS.

**Conclusion:**

The dual-modal US radiomics nomogram revealed superior discrimination accuracy and considerably decreased unnecessary FNAB rates in benign and malignant TR4-5 TNs. It could guide further examination or treatment options.

**Supplementary Information:**

The online version contains supplementary material available at 10.1186/s40644-024-00661-3.

## Background

Thyroid ultrasound (US) is the first-line imaging choice to detect thyroid nodules (TNs) and differentiate benign TNs from malignant nodules [1]. Over the past few decades, the incidence rates of both TNs and thyroid cancer have increased due to the prevalence of ultrasonography and fine needle aspiration biopsy (FNAB), respectively [2]. However, using ultrasonography to differentiate benign and malignant TNs is strongly operator-dependent and has a great interobserver variation. To fulfill standardized management of TNs, the Committee of the American College of Radiology (ACR) published a white paper in 2017 based on comprehensive scores of five US grayscale features, including internal composition, echogenicity of the solid part, shape, margin, and echogenic foci called ACR Thyroid Imaging Reporting and Data System (TI-RADS, TR) [3]. This risk stratification system presented different risk levels from TR1 to TR5 for classifying TNs and guided whether to undergo FNAB or US follow-up according to their maximum diameter. However, it is a challenging issue to differentiate benign from malignant TR4-5 TNs, as they demonstrate much more complicated features and overlapping compositions, echoes, boundaries, and morphologies than TR1-3 TNs [4]. Moreover, TR4-5 TNs exhibit a broad spectrum of potential malignancy rates (> 5%), which could result in excessive diagnosis or incorrect diagnosis, leading to unnecessary FNAB and thyroid surgery, and ultimately impacting the individual’s quality of life adversely. Therefore, the development of an accurate and noninvasive diagnostic method is expected to improve diagnostic accuracy and decrease unnecessary FNAB for TR4-5 TNs.

Apart from the morphologic information provided by B-mode ultrasound (BMUS), intra-nodular blood flow distribution, and vascular characteristics also have an important role in differentiating benign and malignant TNs [5]. As a noninvasive ultrasonic technology for evaluating microvascular perfusion in TNs in daily clinical practice, contrast-enhanced ultrasound (CEUS) is commonly served as an important complement to BMUS and has been demonstrated to improve the diagnostic specificity in combination with grayscale US in the evaluation of TNs [6]. It was also reported in a meta-analysis that both qualitative and quantitative CEUS showed a good performance in differentiating between benign and malignant TNs [7]. Previous studies have reported heterogeneous hypo-enhancement is the most common predictor of malignancy, while homogeneous iso/hyperenhancement or a ring enhancement pattern likely indicates a benign nodule on CEUS [8]. However, overlapping characteristics of CEUS criteria of benign and malignant TNs and observer variability still exist [9]. In fact, no single ultrasonic mode is perfect with sufficient sensitivity or specificity. Furthermore, medical professionals assess the danger of TNs and subsequently determine the course of action based on a thorough evaluation of clinical and US data. Hence, these complementary US techniques should be in conjunction with other clinical data to improve diagnostic accuracy in evaluating TNs.

In recent times, radiomics analysis of medical imaging has emerged as a popular research area in the field of artificial intelligence, owing to its ability to overcome the inherent subjectivity associated with the traditional visual interpretation of medical images and transform imaging data into objective quantitative biomarkers using cutting-edge computational techniques [10]. Prior studies have demonstrated that the radiomic features of US can aid in distinguishing between benign and malignant TNs [11–13]. However, referring to differentiating benign and malignant TR4-5 TNs, the integrated system of combining deep learning network and traditional machine learning radiomics network developed by Wang et al. only got an area under the receiver operating characteristic curve (AUC) of 0.800 and an accuracy of 76.8% in the test set [4]. Wu et al. found that the performance of deep learning convolutional neural networks was also weaker in the combined TR4 and TR5 datasets than separated TR4 dataset or TR5 dataset, with an AUC of 0.829 and accuracy of 78.4% in the independent external test set, which might be correlated with a more complex task when mixing different imaging features in TR4 and TR5 TNs [14]. As differentiating benign and malignant TR4-5 TNs was a tough task for radiologists, our study mainly focused on how to improve diagnostic accuracy in differentiating benign nodules from malignant TR4-5 TNs, which had much more complicated characteristics than TR1-3 TNs.

A nomogram is an individualized evidence-based graphical model used to predict clinical outcomes in a concise and objective manifestation. Some studies have shown that nomograms incorporating clinical and US risk factors such as age, echogenicity, shape, margin, and echogenic foci help in predicting malignant TNs [15, 16]. We assumed that a nomogram involving clinicopathological features, visual evaluation, and radiomics-derived data of BMUS and CEUS images to obtain better predictive performance for ACR TI-RADS 4–5 TNs. To the best of our knowledge, no previous studies have examined whether a nomogram including CEUS radiomics traits could more effectively distinguish benign and malignant ACR TI-RADS 4–5 TNs. Therefore, this study was designed to establish and validate a dual-modal US radiomics nomogram integrating BMUS and CEUS imaging to improve the accuracy of diagnosis and reduce unnecessary FNAB rates in ACR TI-RADS 4 and 5 TNs.

## Methods

### Patients

Between December 2019 and November 2022, consecutive patients with TNs were collected. This retrospective study was approved by the hospital Institutional Review Board and the informed consent for using patient data was waived. However, informed consent for the CEUS examinations was obtained from all patients.

The inclusion criteria were as follows: (1) ACR TI-RADS 4 and 5 category TNs; (2) US data of BMUS and CEUS and basic clinical data were complete; (3) the nodule had definite surgical pathological or FNAB results.

The exclusion criteria were as follows: (1) nodules with benign cytological findings not validated by two repeat FNABs or experiencing enlargement on US or an alteration of ACR TI-RADS classification over a minimum of six months’ surveillance; (2) the patients who had a history of FNAB or ablation; (3) the nodule is too large to reveal the whole lesion or has no surrounding normal parenchyma as a reference.

Ultimately, a total of 312 nodules from 269 patients (mean age, 40.17 ± 11.31 years, range, 18–69 years; 53 men and 216 women) were enrolled in our study. All nodules were randomly divided into the training group (*n* = 219) and the validation group (*n* = 93) with a ratio of 7:3. More detailed inclusion and exclusion steps were presented in Fig. S1 in the Supplemental materials.

### Clinicopathologic information and dual-modal US images acquisition

All patients’ baseline clinical-pathologic information, including age, sex, surgical pathologies or FNAB results, and US diagnostic reports (largest diameter and location of the target nodule) were collected from medical records. All TNs’ BMUS and CEUS images were acquired with the same US device (Canon Aplio i800, Canon Medical Systems) using a 5–18 MHz linear transducer. The operation and diagnosis of TNs were independently performed by one radiologist with more than 20 years of experience in thyroid US diagnosis and 5 years of experience in thyroid CEUS. Images of the maximum cross-section of each target nodule on BMUS were preserved, and video clips of BMUS images were also obtained. Then the focus was adjusted to the lower edge of the target nodule and CEUS mode was switched. Continuous cine was stored by injecting SonoVue (Bracco) through the elbow vein. We then exported all the static images and dynamic clips of BMUS and CEUS to the USB.

### Qualitative analysis of BMUS and CEUS

All BMUS and CEUS images and dynamic videos were evaluated independently by two radiologists (with > 8 years of experience in thyroid US diagnosis and 5 years of experience in thyroid CEUS) who were blinded to all the clinicopathological information of TNs. When there were any discrepancies, they negotiated to reach a consensus.

For BMUS, the composition (mixed cystic and solid, solid or almost completely solid), echogenicity (isoechoic, hyperechoic, hypoechoic relative to adjacent thyroid parenchyma, very hypoechoic relative to adjacent strap muscles), shape (wider-than-tall, taller-than-wide), margin (smooth or ill-defined, lobulated or irregular, extra-thyroidal extension), echogenic foci (none or large comet-tail artifacts, macrocalcifications, peripheral calcifications, punctate echogenic foci), TI-RADS score and risk level were recorded of each TN according to the lexicon of 2017 ACR TI-RADS [3]. TR4 TNs with a maximal diameter ≥ 15 mm, and TR5 TNs with a maximal diameter ≥ 10 mm were recommended for FNAB.

For CEUS, each nodule’s enhancement direction (scattered, centripetal, centrifugal), enhancement pattern (homogeneous, heterogeneous), peak intensity (nonenhancement, hypo-enhancement, iso-enhancement, hyper-enhancement relative to adjacent thyroid parenchyma at peak), ring enhancement (absent, present) were recorded.

### Radiomics analysis of dual-modal US images

#### Region of interest (ROI) segmentation

Each target nodule was manually segmented around the nodule outline on the BMUS image of the largest cross-section using ITK-SNAP 3.8.0. For the TN segmentation on the CEUS image, firstly the offline external perfusion analysis software (VueBox®) was used to generate the CEUS quantitative parameters including the peak enhancement and time to peak. Then the single frame matching the moment of peak enhancement of the CEUS clips of the TN was chosen to be representative of the whole CEUS process for analysis as there was a significant difference in intra-nodular peak enhancement of CEUS between benign and malignant TNs [17]. On the dual-mode CEUS image, the ROI of the nodule on the BMUS image was segmented first, then copied and mapped to the corresponding CEUS image due to an indefinite borderline of the nodule on the CEUS image. The detailed TIC analysis procedures of CEUS videos are presented in Supplementary A1.

All the TNs’ manual delineations on the BMUS and CEUS images were conducted by a radiologist (Doctor A) with 10 years of experience in thyroid US imaging who was blinded to the clinicopathologic result of TNs. Then at a one-week interval, fifty TNs with the BMUS and CEUS images were randomly selected and independently segmented by Doctor A and another radiologist (Doctor B) with 8 years of experience in thyroid US imaging to evaluate the intra-observer and inter-observer reproducibility of the extracted radiomics features, respectively. Features with an interclass correlation coefficient (ICC) that was greater than 0.75 were considered to have a high consistency.

### Radiomics feature extraction, selection, and radiomics score (Rad-score) building

Open-source software (Pyradiomics; version 3.0.1, http://pyradiomics.readthedocs.io) was applied to extract textural, morphological, intensity, and wavelet features automatically from each ROI of the BMUS and CEUS images. After the BMUS radiomics feature set and the CEUS radiomics feature set were obtained, dimensionality reduction and TR4-5 TNs status-related radiomics feature selection were performed on the feature data extracted from BMUS and CEUS images in the training set. First, insignificant characteristics with *P*-values ≥ 0.05 were removed using univariate analyses. Then, variables that were highly correlated (with a Spearman’s correlation coefficient of ≥ 0.8) were eliminated to avoid redundancy. Finally, the least absolute shrinkage and selection operator (LASSO) logistic regression algorithm using ten-fold cross-validations was applied to select the remaining most predictive TR4-5 TNs status-related features from the training cohort.

The Rad-score was built via a linear amalgamation of the selected characteristics, with weighting determined by the LASSO algorithm. The equation for the BMUS and CEUS Rad-scores were constructed using the chosen respective features in the training and validation groups, respectively, and the possible association between the Rad-scores and the characteristics of TR4-5 TNs from BMUS and CEUS images was evaluated using a Mann-Whitney U test.

### Dual-modal US radiomics nomogram construction

Differences in clinical and dual-modal US risk factors associated with benign and malignant TR4-5 TNs were assessed using univariate analyses. Then a multivariate logistic regression analysis involving the Rad-scores and significant clinical and US risk factors was conducted, employing a stepwise backward selection approach with a liberal *P*-value threshold of < 0.05 as the retention standard to identify the ultimate significant predictors for assessing TR4-5 TNs. Finally, a dual-modal US radiomics nomogram was built with Rad-scores, and clinical and US characteristics in the training cohort. For comparison, another two predictive models based on independent clinical combined US risk factors, and dual-modal US Rad-score were established using the same method, respectively.

### Performance evaluation

The calibration curve and Hosmer-Lemeshow test were plotted to assess the calibration effect of the dual-modal US radiomics nomogram. The discriminative performance of the dual-modal US radiomics nomogram was evaluated using the AUC. Then the performance of the dual-modal US radiomics nomogram was tested in the validation cohort using the calibration curve and AUC. AUCs of the dual-modal US radiomics nomogram and another two predictive models were compared in the training, validation, and entire cohorts. A decision curve analysis (DCA) was used to evaluate the clinical usefulness of the dual-modal US radiomics nomogram by guiding FNAB at different thresholds by quantifying the net benefits in the entire cohort. The predictive importance of the dual-modal US radiomics nomogram was assessed by the index integrated discrimination improvement (IDI) and the net reclassification improvement (NRI). For clinical use, the dual-modal US radiomics nomogram predicting the probability of malignancy of each nodule (defined as Nomo-score) was calculated based on the nomogram algorithm. Then the optimal Nomo-score cutoff value was assessed by maximizing the Youden index. The performance of the optimal Nomo-score cutoff value was assessed by accuracy, sensitivity, specificity, predictive values, and likelihood ratios.

If the predictive models yielded a positive result, the TNs were recommended for FNAB, while those with a negative result were not recommended. The rate of unnecessary FNAB was calculated as the proportion of benign TNs among the recommended biopsied TNs.

### Statistical analyses

The statistical analyses were performed with R version 3.6.1, SPSS version 27.0, and MedCalc version 20.027. In the univariate analysis, Student’s t-test (for normally distributed characteristics) or Mann-Whitney U test (for non-normally distributed characteristics) was used for continuous variables and a chi-square test or Fisher’s exact test (categorical variables) was used for categorical variables as appropriate. The DeLong test was used to compare differences in the AUC of three different models in the training, validation, and entire cohorts. All the statistically significant differences were a two-sided *P* value < 0.05. R software and descriptions of the associated step algorithm are provided in the Supplemental materials (Table S1).

## Results

### Clinical and dual-modal US characteristics of TNs

**Fig. 1:**
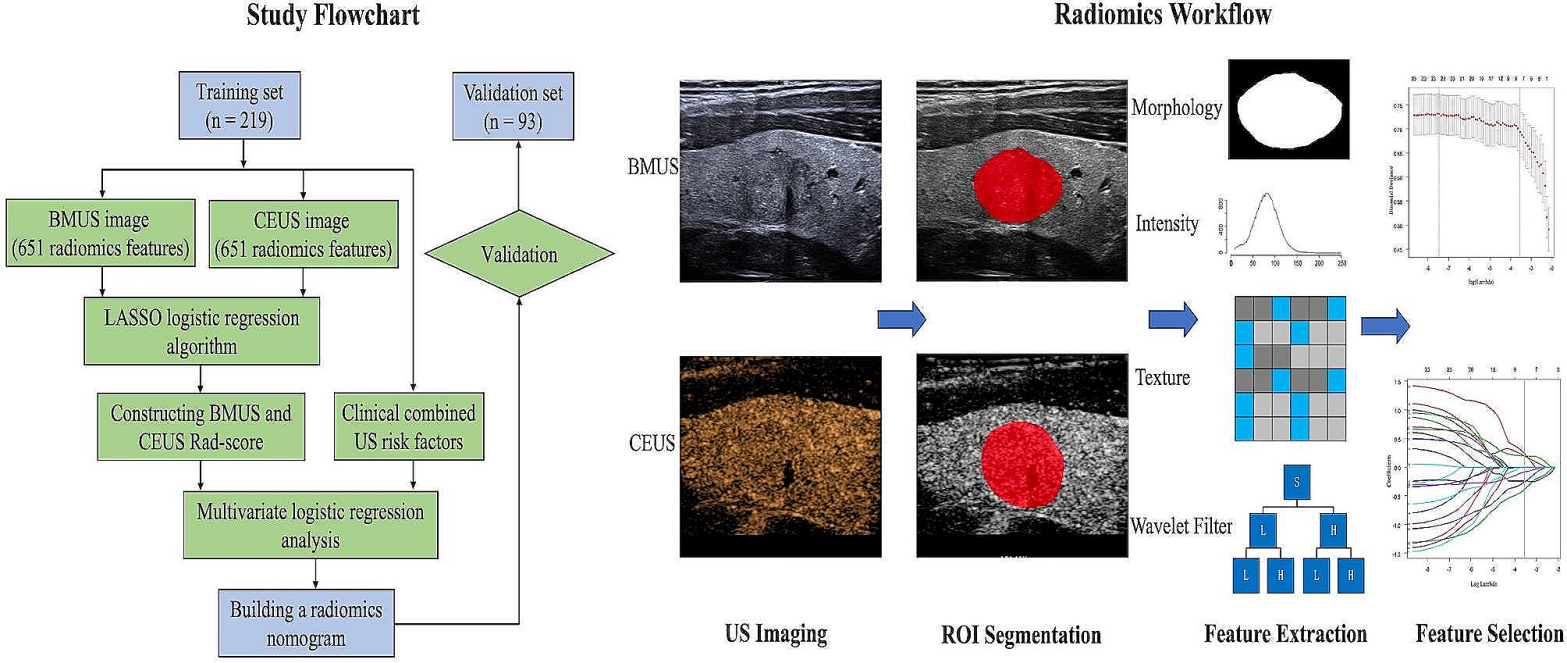
The study flowchart and ultrasound radiomics workflow of the present study. BMUS = B-mode ultrasound, CEUS = contrast-enhanced ultrasound, LASSO = least absolute shrinkage and selection operator, Rad-score = radiomics score, US = ultrasound, ROI = region of interest

The study flowchart and radiomics workflow are presented in Fig. 1. Of the 312 TNs, 219 (70.2%) nodules were malignant (containing 214 papillary thyroid carcinomas, 3 follicular carcinomas, and 2 medullary carcinomas). Among the 93 benign nodules, 65 (69.9%) were confirmed by excised surgeries, including 47 nodular goiter, 1 adenomatous goiter, 1 diffuse toxic goiter, 10 follicular adenomas, 3 oxyphilic adenomas, and 3 subacute thyroiditis. For the remaining 28 (30.1%) benign nodules, 7 were determined by the concordant benign cytological results of twice FNABs, and 21 were validated by the initial benign cytological results of FNAB and a decreased or stable size on at least six months of US follow-up. The detailed clinical and US features on the training and validation sets are summarized in Tables 1 and 2. There were no significant differences in the remaining clinical and US characteristics between the training and validation datasets, except for composition (*P* = 0.024). In the training cohort, univariate analyses for each clinical and US characteristic revealed that age, tumor size, primary site, composition, shape, margin, echogenic foci, enhancement direction, enhancement pattern, and ring enhancement were significantly different in differentiating between benign and malignant TR4-5 TNs. Then a multivariate logistic regression analysis based on the above ten predictive risk factors demonstrated that age, shape, echogenic foci, enhancement direction, and ring enhancement were independent predictors of the nature of TR4-5 TNs. Finally, a clinical combined with US model was constructed based on the final five predictive risk factors for differentiating the nature of TR4-5 TNs. Table 3 displays the performance of the ACR TI-RADS for estimating the malignant risk of TR 4–5 TNs.

**Table 1:** Clinical and ultrasound characteristics in the training and validation cohorts

Characteristic	Training (*n* = 219)	Validation (*n* = 93)	P value
**Sex**			0.707
Male	43 (19.6)	20 (21.5)	
Female	176 (80.4)	73 (78.5)	
**Age (years)**			0.795
Mean ± SD	40.33 ± 11.16	39.98 ± 10.77	
Range	18–69	18–64	
Tumor size, median (IQR)	10 (8–16)	12 (7–23)	0.111
**Primary site**			0.289
Right	117 (53.4)	45 (48.4)	
Left	96 (43.8)	42 (45.2)	
Isthmus	6 (2.7)	6 (6.5)	
**Pathology**			0.845
Benign	66 (30.1)	27 (29.0)	
Malignant	153 (69.9)	66 (71.0)	
**Composition**			0.024
Mixed cystic and solid	22 (10.0)	18 (19.4)	
Solid or almost completely solid	197 (90.0)	75 (80.6)	
**Echogenicity**			0.234
Hyperechoic or isoechoic	4 (1.8)	0 (0.0)	
Hypoechoic	212 (96.8)	92 (98.9)	
Very hypoechoic	3 (1.4)	1 (1.1)	
**Shape**			0.087
Wider-than-tall	104 (47.5)	54 (58.1)	
Taller-than-wide	115 (52.5)	39 (41.9)	
**Margin**			0.173
Smooth or ill-defined	39 (17.8)	10 (10.8)	
Lobulated or irregular	177 (80.8)	80 (86.0)	
Extra-thyroidal extension	3 (1.4)	3 (3.2)	
**Echogenic foci**			0.245
None or large comet-tail artifacts	77 (35.2)	30 (32.3)	
Macrocalcifications	20 (9.1)	4 (4.3)	
Punctate echogenic foci	123 (55.7)	59 (63.4)	
**TI-RADS risk level**			0.604
TR4	46 (21.0)	22 (23.7)	
TR5	173 (79.0)	71 (76.3)	
**Enhancement direction**			0.724
Scattered	57 (26.0)	26 (28.0)	
Centripetal or centrifugal	162 (74.0)	67 (72.0)	
**Enhancement pattern**			0.201
Homogeneous	44 (20.1)	13 (14.0)	
Heterogeneous	175 (79.9)	80 (86.0)	
**Peak intensity**			0.955
None- or iso-enhancement	43 (19.6)	18 (19.4)	
Hypo- or Hyper-enhancement	176 (80.4)	75 (80.6)	
**Ring enhancement**			0.106
Absent	192 (87.7)	75 (80.6)	
Present	27 (12.3)	18 (19.4)	
BMUS Rad-score, median (IQR)	0.93 (0.56–1.26)	0.90 (0.49–1.24)	0.715
CEUS Rad-score, median (IQR)	1.05 (0.60–1.34)	1.03 (0.50–1.34)	0.624

*Note*: Data is the number of thyroid nodules with percentages in parentheses unless otherwise noted. ACR TI-RADS = American College of Radiology Thyroid Imaging Reporting and Data System, SD = standard deviation, IQR = interquartile range, BMUS = B-mode ultrasound, CEUS = contrast-enhanced ultrasound

**Table 2:** Clinical and ultrasound characteristics predicting malignancy of ACR TI-RADS 4 and 5 thyroid nodules

Characteristic	Training (*n* = 219)			Validation (*n* = 93)		
	Benign (*n* = 66)	Malignant (*n* = 153)	P value	Benign (*n* = 27)	Malignant (*n* = 66)	P value
**Sex**			0.468			0.032
Male	11 (16.7)	32 (20.9)		2 (7.4)	18 (27.3)	
Female	55 (83.3)	121 (79.1)		25 (92.6)	48 (72.7)	
**Age (years)**			0.029			0.620
Mean ± SD	42.83 ± 10.94	39.25 ± 11.11		40.85 ± 11.24	39.62 ± 10.64	
Range	19–69	18–67		21–64	18–60	
Tumor size (mm), median (IQR)	12 (8–20)	10 (8–15)	0.048	22 (17–29)	10 (6–15)	< 0.001
**Primary site**			0.033			0.589
Right	31 (47.0)	86 (56.2)		15 (55.6)	30 (45.5)	
Left	35 (53.0)	61 (39.9)		11 (40.7)	31 (47.0)	
Isthmus	0 (0)	6 (3.9)		1 (3.7)	5 (7.6)	
**Composition**			0.032			0.029
Mixed cystic and solid	11 (16.7)	11 (7.2)		9 (33.3)	9 (13.6)	
Solid or almost completely solid	55 (83.3)	142 (92.8)		18 (66.7)	57 (86.4)	
**Echogenicity**			0.414			0.406
Hyperechoic or isoechoic	1 (1.5)	3 (2.0)		0 (0.0)	0 (0.0)	
Hypoechoic	63 (95.5)	149 (97.4)		27 (100.0)	65 (98.5)	
Very hypoechoic	2 (3.0)	1 (0.7)		0 (0.0)	1 (1.5)	
**Shape**			< 0.001			0.014
Wider-than-tall	47 (71.2)	57 (37.3)		21 (77.8)	33 (50.0)	
Taller-than wide	19 (28.8)	96 (62.7)		6 (22.2)	33 (50.0)	
**Margin**			< 0.001			0.001
Smooth or ill-defined	27 (40.9)	12 (7.8)		8 (29.6)	2 (3.0)	
Lobulated or irregular	39 (59.1)	138 (90.2)		19 (70.4)	61 (92.4)	
Extra-thyroidal extension	0 (0)	3 (2)		0 (0.0)	3 (4.5)	
**Echogenic foci**			< 0.001			0.298
None or large comet-tail artifacts	35 (53.0)	42 (27.5)		11 (40.7)	19 (28.8)	
Macrocalcifications	8 (12.1)	12 (7.8)		2 (7.4)	2 (3.0)	
Punctate echogenic foci	23 (34.8)	99 (64.7)		14 (51.9)	45 (68.2)	
**TI-RADS risk level**			< 0.001			< 0.001
TR4	28 (42.4)	18 (11.8)		15 (55.6)	7 (10.6)	
TR5	38 (57.6)	135 (88.2)		12 (44.4)	59 (89.4)	
**Enhancement direction**			< 0.001			< 0.001
Scattered	31 (47.0)	26 (17.0)		15 (55.6)	11 (16.7)	
Centripetal or centrifugal	35 (53.0)	127 (83.0)		12 (44.4)	55 (83.3)	
**Enhancement pattern**			0.001			0.430
Homogeneous	22 (33.3)	22 (14.4)		5 (18.5)	8 (12.1)	
Heterogeneous	44 (66.7)	131 (85.6)		22 (81.5)	58 (87.9)	
**Peak intensity**			0.062			0.305
None- or iso-enhancement	18 (27.3)	25 (16.3)		7 (25.9)	11 (16.7)	
Hypo- or Hyper-enhancement	48 (72.7)	128 (83.7)		20 (74.1)	55 (83.3)	
**Ring enhancement**			< 0.001			< 0.001
Absent	47 (71.2)	145 (94.8)		15 (55.6)	60 (90.9)	
Present	19 (28.8)	8 (5.2)		12 (44.4)	6 (9.1)	
BMUS Rad-score, median (IQR)	0.64 (0.15–0.97)	1.05 (0.73–1.35)	< 0.001	0.49 (0.13–1.05)	0.95 (0.65–1.37)	0.001
CEUS Rad-score, median (IQR)	0.58 (0.03–1.06)	1.18 (0.85–1.40)	< 0.001	0.64 (0.07–0.70)	1.15 (0.87–1.40)	< 0.001

*Note*: Data is the number of thyroid nodules with percentages in brackets unless otherwise noted. ACR TI-RADS = American College of Radiology Thyroid Imaging Reporting and Data System, SD = standard deviation, IQR = interquartile range, BMUS = B-mode ultrasound, CEUS = contrast-enhanced ultrasound

**Table 3:** Predictive performance of the ACR TI-RADS for TI-RADS 4 and 5 thyroid nodules

Variable	Value (95% CI)		
	Training cohort (*n* = 219)	Validation cohort (*n* = 93)	Entire cohort (*n* = 312)
Cutoff value	0.586	0.586	0.586
AUC	0.653 (0.588–0.719)	0.669 (0.567–0.772)	0.658 (0.603–0.713)
Sensitivity, %	88.2 (83.0-93.5)	89.4 (81.8–97.0)	88.6 (84.5–92.7)
Specificity, %	42.4 (30.3–54.5)	44.4 (25.9–63.0)	43.0 (33.3–52.7)
PPV, %	78.0 (71.9–84.2)	79.7 (70.6–88.9)	78.5 (73.4–83.7)
NPV, %	60.9 (46.8–75.0)	63.2 (41.5–84.8)	61.5 (49.7–73.4)
PLR	2.771 (1.925–3.988)	3.116 (1.766–5.496)	2.868 (2.111–3.896)
NLR	0.501 (0.347–0.725)	0.462 (0.254–0.842)	0.490 (0.358–0.671)
Accuracy, %	74.4 (68.1–80.1)	76.3 (66.4–84.5)	75.0 (69.8–79.7)

*Note*: ACR TI-RADS = American College of Radiology Thyroid Imaging Reporting and Data System, CI = confidence interval, AUC = the area under the receiver operating characteristic curve, PPV = positive predictive value, NPV = negative predictive value, PLR = positive likelihood ratio, NLR = negative likelihood ratio

### Dual-modal US rad-score building

A set of 651 radiomics features was extracted from the BMUS and CEUS modes of each TN, respectively. After intra-observer and inter-observer reproducibility of the extracted radiomics features were evaluated, 639 out of 651 BMUS features and 630 out of 651 CEUS features were retained. For BMUS, the radiomics features were reduced to 10 features after LASSO regression in the training cohort (Supplementary Fig. S2A, B). Likewise, the CEUS radiomics features were reduced to 7 risk predictors by LASSO algorithm in the training cohort (Supplementary Fig. S2C, D). The Rad-score calculation formulas for BMUS and CEUS are provided in the Supplementary material (Supplementary A2). The BMUS and CEUS Rad-scores were all significantly higher in the malignant TR4-5 nodule group than that in the benign group in both the training and validation cohorts (Table 2). Then, a dual-modal US Rad-score model was constructed based on both BMUS Rad-score and CEUS Rad-score for differentiating benign from malignant TR4-5 TNs.

### Dual-modal US radiomics nomogram construction and evaluation

**Fig. 2:**
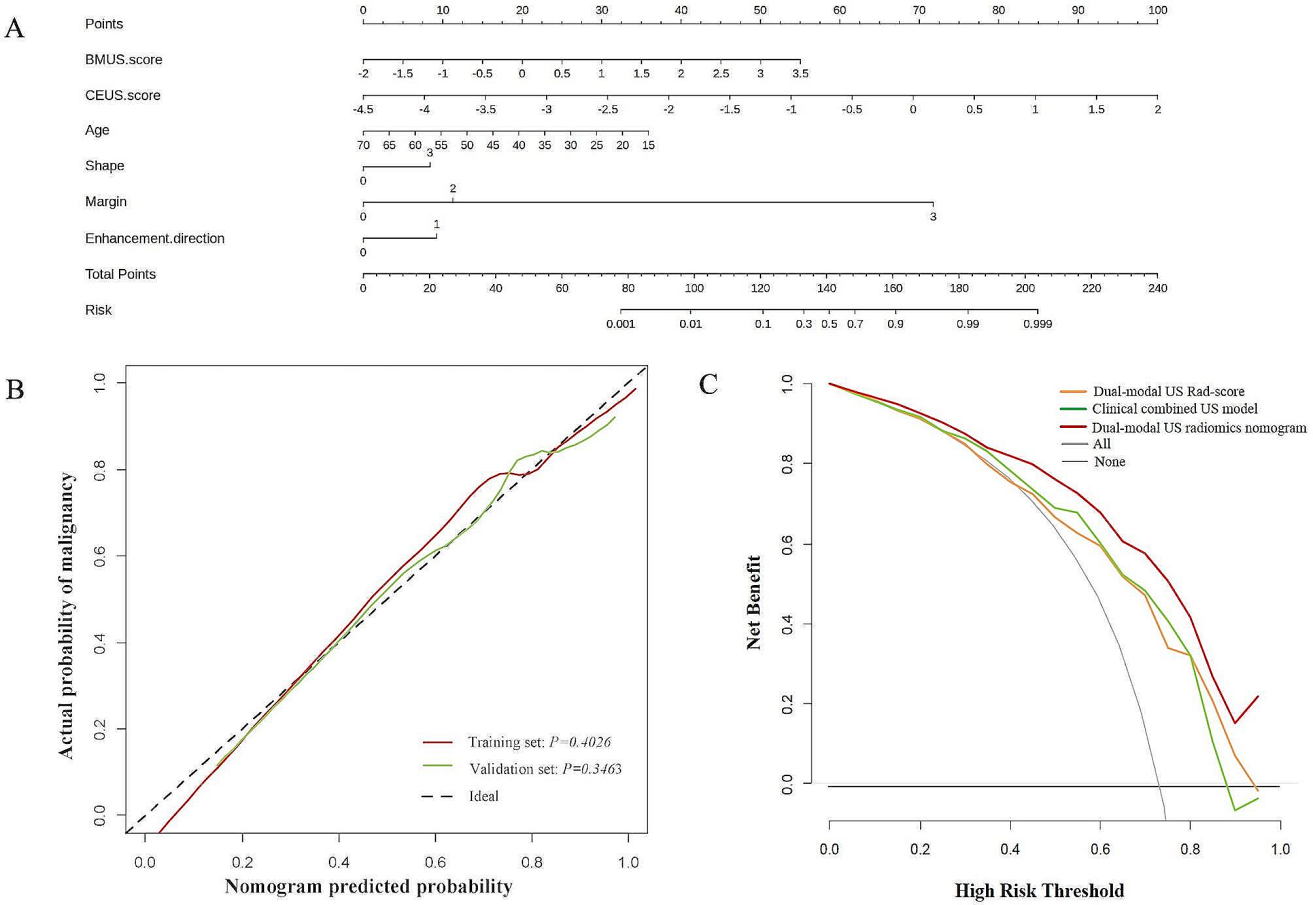
Dual-modal US radiomics nomogram and its predictive performance for TI-RADS 4 and 5 thyroid nodules. (**A**) A dual-modal US radiomics nomogram was constructed with BMUS Rad-score, CEUS Rad-score, age, shape, margin, and enhancement direction for predicting malignancy of TI-RADS 4–5 thyroid nodules. (**B**) Calibration curves of the dual-modal US radiomics nomogram in the training and validation cohorts. The red and green lines represent the actual predictive probabilities of malignancy of the nomogram in the training and validation cohorts, respectively, and the dashed black line represents an ideal prediction. (**C**) A decision curve analysis (DCA) shows the role of three different models in predicting benign and malignant TI-RADS 4–5 thyroid nodules derived from the entire cohort (*n* = 312). The DCA shows that using the dual-modal US radiomics nomogram (red curve) to predict benign and malignant TI-RADS 4–5 thyroid nodules provided a greater benefit than the clinical combined US model (green curve) and dual-modal US Rad-score (orange curve). BMUS = B-mode ultrasound, CEUS = contrast-enhanced ultrasound, US = ultrasound, Rad-score = radiomics score

The BMUS Rad-score, CEUS Rad-score, age, shape, margin, and enhancement direction were identified as independent predictors for the nature of TR4-5 TNs by multivariate logistic regression analysis in the training cohort (Table 4). A dual-modal US radiomics nomogram based on the above independent risk predictors was constructed (Fig. 2A). The Hosmer-Lemeshow test statistic (*P* = 0.403 and 0.346 for the training and validation cohorts, respectively) and calibration curve showed good calibration of the dual-modal US radiomics nomogram for predicting benign and malignant TR4-5 TNs in the training and validation cohorts (Fig. 2B). The DCA curves showed that the dual-modal US radiomics nomogram was more beneficial than the clinical combined with US model or dual-modal US Rad-score model alone at all different threshold probabilities in the entire cohort (Fig. 2C).

**Table 4:** Construction of three different models based on risk factors in the training cohort

Intercept and variable	Dual-modal US radiomics nomogram			Clinical combined with US model			Dual-modal US Rad-score		
	Coef	Odds ratio (95% CI)	P value	Coef	Odds ratio (95% CI)	P value	Coef	Odds ratio (95% CI)	P value
Intercept	-0.648			0.519			-1.475		
Age	-0.072	0.931 (0.895–0.969)	< 0.001	-0.043	0.958 (0.925–0.992)	0.017	NA	NA	NA
Shape	0.307	1.360 (1.038–1.781)	0.026	0.403	1.496 (1.169–1.913)	0.001	NA	NA	NA
Margin	1.238	3.447 (1.309–9.081)	0.012	NA	NA	NA	NA	NA	NA
Echogenic foci	NA	NA	NA	1.303	3.678 (1.715–7.890)	< 0.001	NA	NA	NA
Enhancement direction	1.012	2.751 (1.147-6.600)	0.023	1.480	4.391 (2.002–9.628)	< 0.001	NA	NA	NA
Ring enhancement	NA	NA	NA	-1.303	0.272 (0.099–0.747)	0.012	NA	NA	NA
BMUS Rad-score	1.098	2.997 (1.386–6.481)	0.005	NA	NA	NA	1.317	3.732 (1.876–7.426)	< 0.001
CEUS Rad-score	1.687	5.405 (2.407–12.135)	< 0.001	NA	NA	NA	1.487	4.422 (2.260–8.652)	< 0.001

*Note*: US = ultrasound, Rad-score = radiomics score, ACR TI-RADS = American College of Radiology Thyroid Imaging Reporting and Data System, Coef = coefficient, CI = confidence interval, NA = not available, BMUS = B-mode ultrasound, CEUS = contrast-enhanced ultrasound

**Fig. 3:**
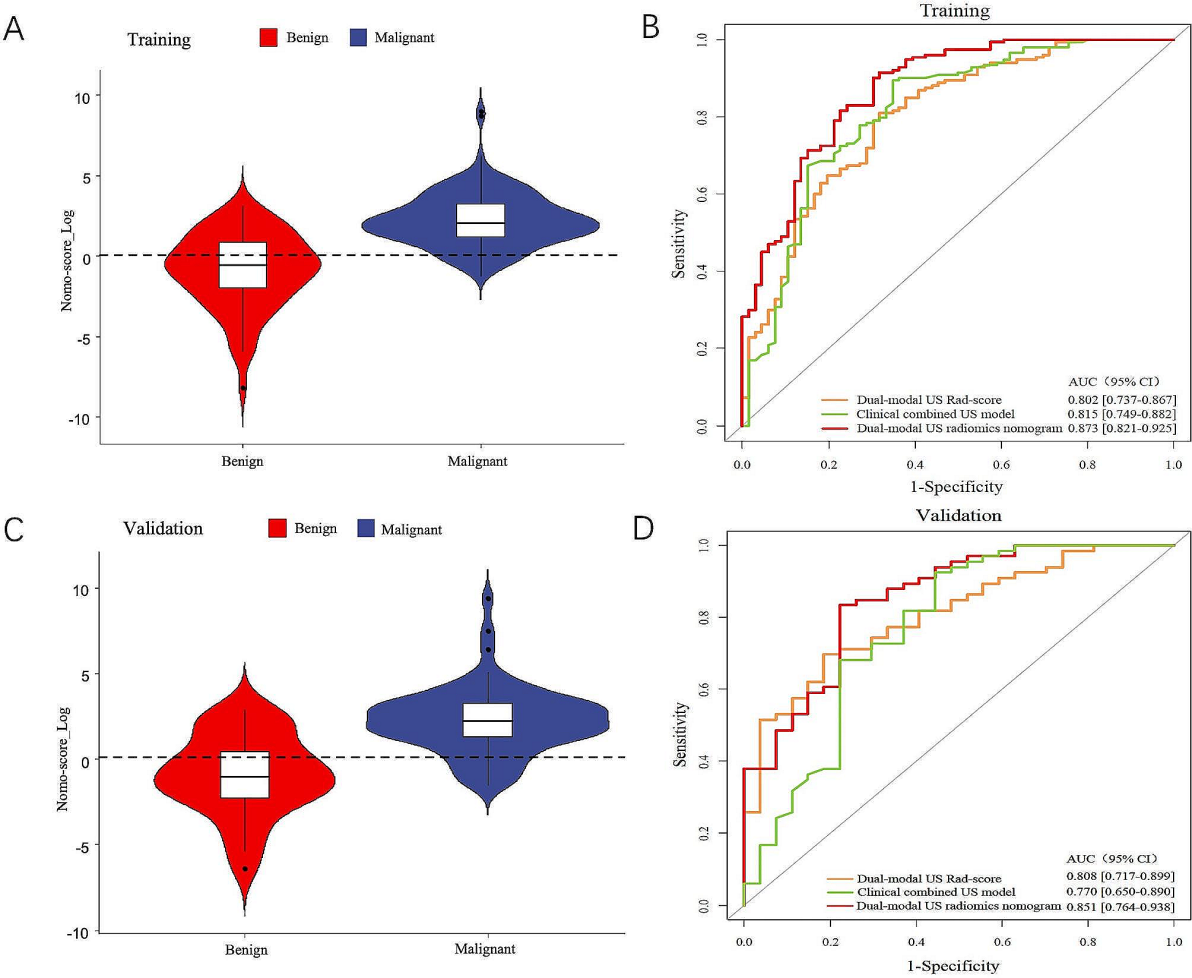
Differential diagnostic accuracy of dual-modal US radiomics nomogram for TI-RADS 4 and 5 thyroid nodules. The violin plot shows that the dual-modal US radiomics nomogram performed well in predicting benign and malignant TI-RADS 4–5 thyroid nodules in both the training (**A**) and validation (**C**) cohorts. The receiver operating characteristic curves of the dual-modal US radiomics nomogram, clinical combined US model, and the dual-modal US Rad-score model are displayed in the training (**B**) and validation (**D**) cohorts, respectively. US = ultrasound, Rad-score = radiomics score, AUC = the area under the receiver operating characteristic curve, CI = confidence interval

The optimal cutoff value of the nomogram score to differentiate benign and malignant TR4-5 TNs was determined to be 0.524 by maximizing the Youden index. The performance of the dual-modal US radiomics nomogram to predict the nature of TR4-5 TNs using the recommended cutoff value is summarized in Table 5. An AUC of 0.873 (95% confidence interval (CI), 0.821–0.925) for the training cohort (Fig. 3A and B) and 0.851 (95% CI, 0.764–0.938) for the validation cohort (Fig. 3C and D) showed good discrimination ability of the dual-modal US radiomics nomogram. Moreover, the dual-modal US radiomics nomogram had better discrimination than the clinical combined with US model and the dual-modal Rad-score model in the training cohort (AUC 0.873 vs. 0.815, *P* = 0.032, 0.873 vs. 0.802, *P* = 0.006) and validation cohort (AUC 0.851 vs. 0.770, *P* = 0.047, 0.851 vs. 0.808, *P* = 0.196) (Table 6). Furthermore, compared with the clinical combined with US prediction model which only incorporated the independent clinical and US risk predictors, the addition of the dual-modal US Rad-score significantly improved the NRI and IDI, implying that dual-modal US Rad-score could be a rather valuable marker for the nature of TR4-5 TNs prediction (Table 7).

**Table 5:** Predictive performance of dual-modal ultrasound radiomics nomogram for ACR TI-RADS 4 and 5 thyroid nodules

Variable	Value (95% CI)		
	Training cohort (*n* = 219)	Validation cohort (*n* = 93)	Entire cohort (*n* = 312)
Cutoff value	0.524	0.524	0.524
AUC	0.873 (0.821–0.925)	0.851 (0.764–0.938)	0.867 (0.822–0.911)
Sensitivity, %	90.2 (85.0-94.78)	86.4 (77.3–93.9)	89.0 (84.9–93.2)
Specificity, %	69.7 (59.1–80.3)	66.7 (48.2–81.5)	68.8 (59.1–78.5)
PPV, %	87.3 (82.2–92.5)	86.4 (78.1–94.6)	87.1 (82.7–91.5)
NPV, %	75.4 (64.6–86.2)	66.7 (48.9–84.5)	72.7 (63.4–82.0)
PLR	3.552 (2.280–5.534)	2.623 (1.614–4.264)	3.104 (2.233–4.315)
NLR	0.168 (0.109–0.259)	0.150 (0.067–0.334)	0.171 (0.117–0.249)
Accuracy, %	84.0 (78.5–88.6)	80.7 (71.2–88.1)	83.0 (78.4–87.0)

*Note*: ACR TI-RADS = American College of Radiology Thyroid Imaging Reporting and Data System, CI = confidence interval, AUC = the area under the receiver operating characteristic curve, PPV = positive predictive value, NPV = negative predictive value, PLR = positive likelihood ratio, NLR = negative likelihood ratio

**Table 6:** Comparison of the AUCs for three different models in the training, validation, and entire cohorts

	Training cohort		Validation cohort		Entire cohort	
	AUC	P value	AUC	P value	AUC	P value
Dual-modal US radiomics nomogram vs. Clinical combined with US model	0.873 vs. 0.815	0.032	0.851 vs. 0.770	0.047	0.867 vs. 0.801	0.003
Dual-modal US radiomics nomogram vs. Dual-modal US Rad-score	0.873 vs. 0.802	0.006	0.851 vs. 0.808	0.196	0.867 vs. 0.803	0.002
Clinical combined with US model vs. Dual-modal US Rad-score	0.815 vs. 0.802	0.773	0.770 vs. 0.808	0.520	0.801 vs. 0.803	0.964

*Note*: AUC = the area under the receiver operating characteristic curve, US = ultrasound, Rad-score = radiomics score

**Table 7:** Predictive value of the dual-modal ultrasound radiomics scores in terms of NRI and IDI

Variable	Training cohort			Validation cohort			Entire cohort		
	Categorical NRI (95% CI)	Continuous NRI (95% CI)	IDI (95% CI)	Categorical NRI (95% CI)	Continuous NRI (95% CI)	IDI (95% CI)	Categorical NRI (95% CI)	Continuous NRI (95% CI)	IDI (95% CI)
BMUS Rad-score	0.093 (-0.026-0.212)	0.532 (0.253–0.812)	0.056 (0.020–0.092)	0.1633 (-0.057-0.384)	0.2929 (-0.151-0.737)	0.083 (0.015–0.150)	0.114 (0.008–0.2197)	0.461 (0.224–0.698)	0.064 (0.032–0.096)
*P* value	0.125	0.002	0.002	0.147	0.196	< 0.05	0.035	< 0.001	< 0.001
CEUS Rad-score	0.2338 (0.091–0.377)	0.6851 (0.411–0.959)	0.122 (0.064–0.180)	0.244 (-0.001-0.489)	0.983 (0.586–1.380)	0.148 (0.058–0.237)	0.237 (0.113–0.361)	0.772 (0.546–0.999)	0.130 (0.081–0.178)
*P* value	0.001	< 0.05	< 0.001	0.05	< 0.05	0.001	< 0.001	< 0.001	< 0.001
BMUS + CEUS Rad-score	0.247 (0.096–0.398)	0.759 (0.488–1.029)	0.152 (0.089–0.216)	0.303 (0.070–0.536)	0.788 (0.372–1.203)	0.176 (0.077–0.276)	0.263 (0.135–0.390)	0.767 (0.540–0.994)	0.159 (0.106–0.213)
*P* value	0.001	< 0.05	< 0.05	< 0.05	< 0.001	< 0.001	< 0.001	< 0.001	< 0.001

*Note*: NRI = net reclassification improvement, IDI = index integrated discrimination improvement, CI = confidence interval = BMUS = B-mode ultrasound, CEUS = contrast-enhanced ultrasound

**Fig. 4:**
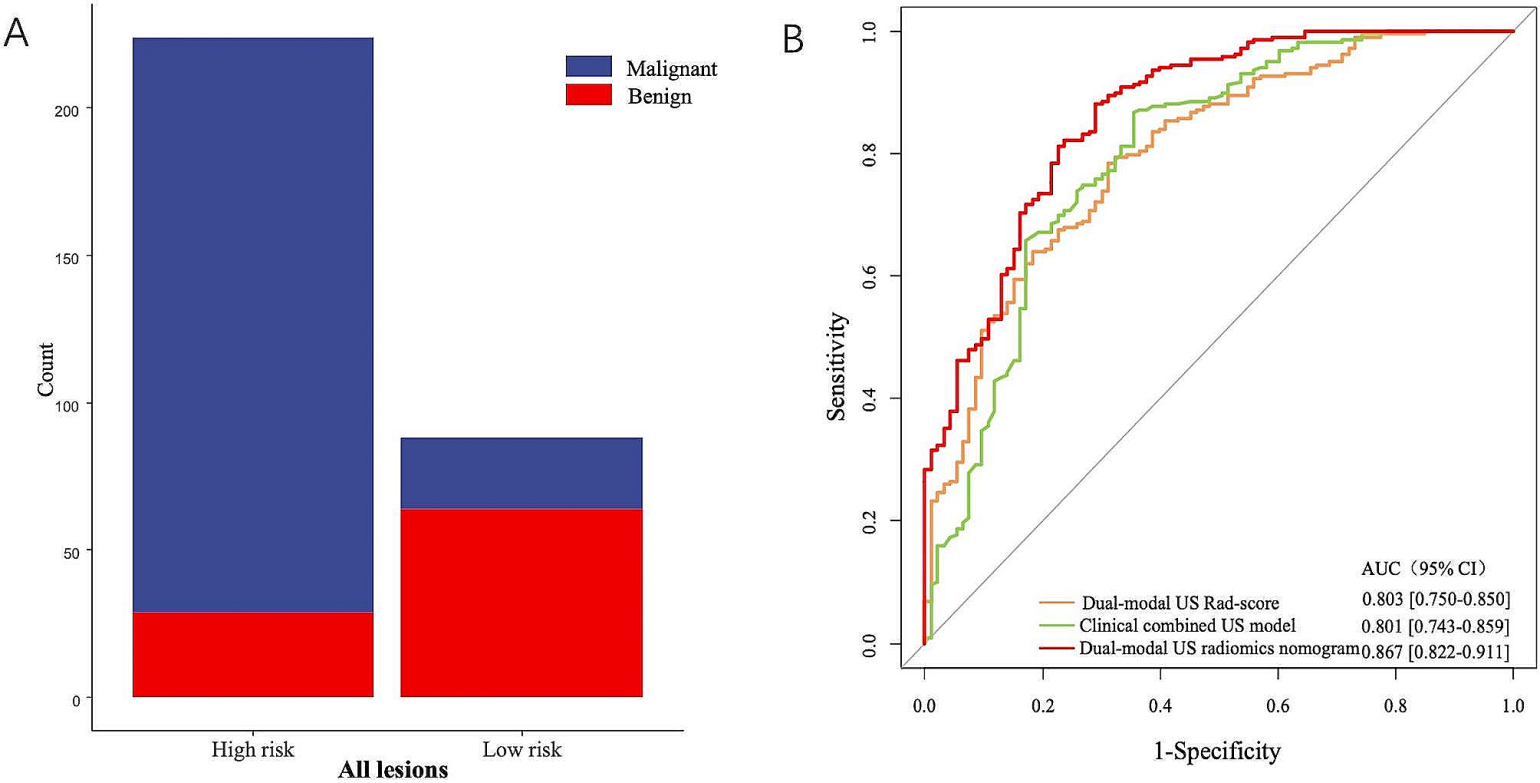
Performance of dual-modal US radiomics nomogram in all 312 TI-RADS 4 and 5 thyroid nodules. (**A**) The risk-classification performance of the dual-modal US radiomics nomogram. (**B**) The ROC curve analyses of the three different models. US = ultrasound, Rad-score = radiomics score, AUC = the area under the receiver operating characteristic curve, CI = confidence interval

**Fig. 5:**
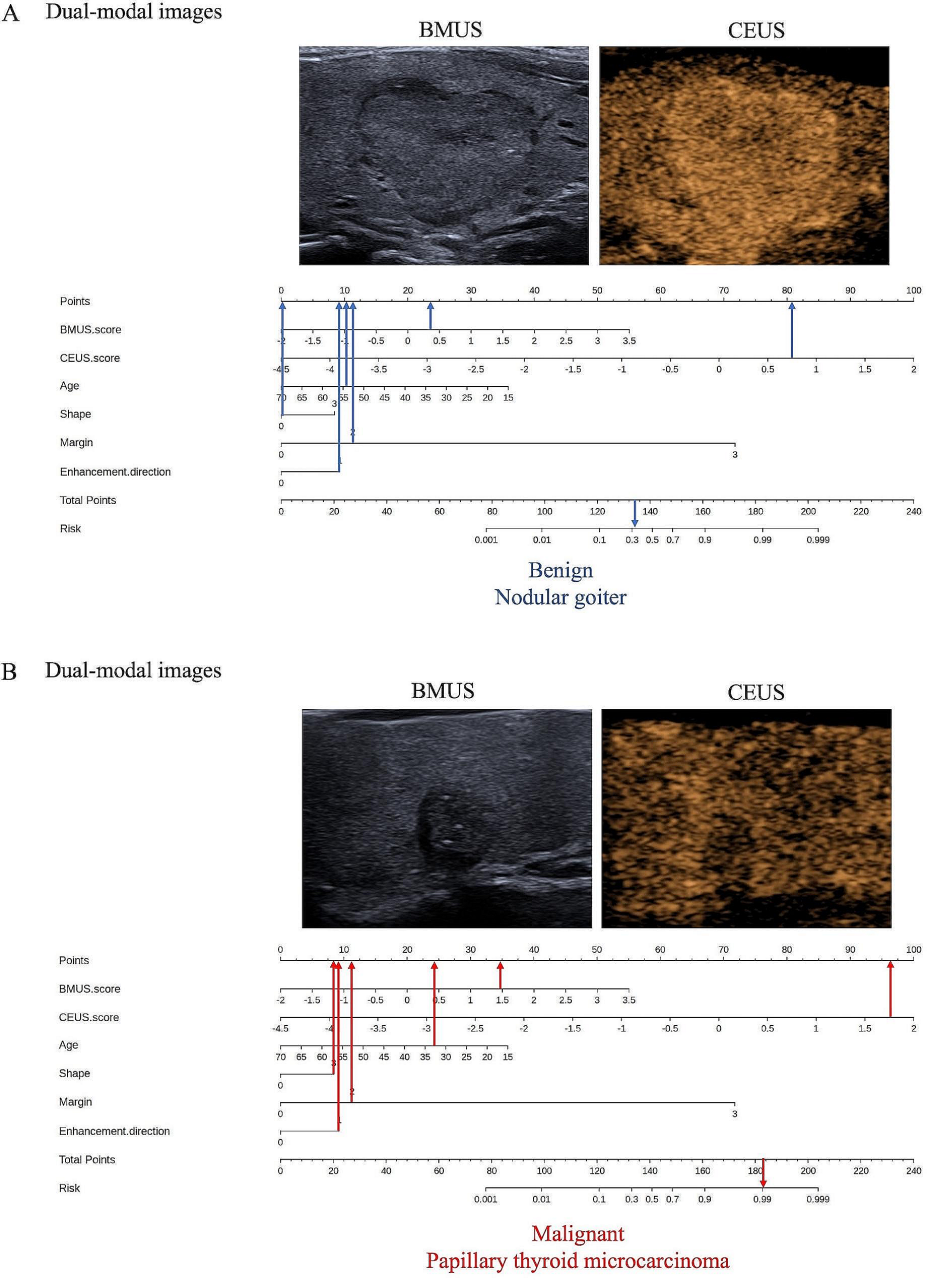
Two illustrative examples to present the clinical utilization of the nomogram as diagrams. (**A**) The blue arrows demonstrated that a 54-year-old patient (point: 10.25) has a thyroid nodule which has an aspect ratio < 1 (point: 0), lobulated margin (point: 11.25), centripetal enhancement direction (point: 9), BMUS radiomics score of 0.354 (point: 23), and CEUS radiomics score of 0.715 (point: 80.5). This thyroid nodule got a total point of 134, corresponding to the malignancy probability (defined as Nomo-score) of 0.339. Therefore, this thyroid nodule was predicted as benign by the nomogram according to the optimal cutoff value of 0.524 and was eventually pathologically confirmed as a nodular goiter. (**B**) The red arrows showed that a 33-year-old (point: 24) patient has a thyroid nodule which has an aspect ratio > 1 (point: 8), irregular margin (point: 11), centripetal enhancement direction (point: 9), BMUS radiomics score of 1.468 (point: 34.75), and CEUS radiomics score of 1.752 (point: 96.25). This thyroid nodule got a total point of 183, referring to a Nomo-score of 0. 991. The nomogram eventually produced an accurate result consistent with the pathology outcome of papillary thyroid carcinoma. BMUS = B-mode ultrasound, CEUS = contrast-enhanced ultrasound

In addition, we further assessed the performance of the dual-mode US radiomics nomogram in all TR4-5 TNs (*n* = 312). All the TR4-5 TNs were classified into low-risk and high-risk subgroups according to the best Nomo-score cutoff value (0.524). The results demonstrated that the high-risk group had a greater proportion of malignant TNs in all TR4-5 TNs (Fig. 4A). The dual-modal US radiomics nomogram yielded a more favorable discriminatory performance than the clinical combined with US model (AUC 0.867 vs. 0.801, *P* = 0.003) and dual-modal US Rad-score model (AUC 0.867 vs. 0.803, *P* = 0.002) in all 312 TR4-5 TNs (Fig. 4B). Figure 5 depicted two illustrative examples of clinical nomogram utilization visualized in diagram.

The unnecessary FNAB rates of the dual-modal US radiomics nomogram and three other predictive models in TR4-5 TNs were calculated and compared in Table 8. Using the dual-modal US radiomics nomogram, the unnecessary FNAB rate decreased from 35.3 to 14.5% (*P* < 0.001) in the training cohort and from 41.5 to 17.7% (*P* = 0.005) in the validation cohorts compared with ACR TI-RADS.

**Table 8:** Comparison of unnecessary FNAB rates of the dual-modal US radiomics nomogram and other models

Diagnostic models	Datasets	No. of recommended FNABs	No. of malignant nodules	No. of benign nodules	Unnecessary FNAB rates (%)	P value*
ACR TI-RADS	Training dataset (*n* = 219)	116	75	41	35.3 (41/116)	< 0.001
	Validation dataset (*n* = 93)	53	31	22	41.5 (22/53)	0.005
	All dataset (312)	169	103	63	37.3 (63/169)	< 0.0001
Dual-modal US radiomics nomogram	Training dataset (*n* = 219)	145	124	21	14.5 (21/145)	NA
	Validation dataset (*n* = 93)	62	51	11	17.8 (11/62)	NA
	All dataset (312)	207	175	32	15.5 (32/207)	NA
Clinical combined with US model	Training dataset (*n* = 219)	160	137	23	14.4 (23/160)	0.980
	Validation dataset (*n* = 93)	65	54	11	16.9(11/65)	0.894
	All dataset (312)	225	191	34	15.1 (34/225)	0.908
Dual-modal US Rad-score	Training dataset (*n* = 219)	158	138	20	12.7 (20/158)	0.648
	Validation dataset (*n* = 93)	66	57	9	13.6 (9/66)	0.515
	All dataset (312)	224	195	29	12.9 (29/224)	0.440

*Note*: *For the comparison of unnecessary FNAB rates between dual-modal US radiomics nomogram and other models. FNAB = fine needle aspiration biopsy, ACR TI-RADS = American College of Radiology Thyroid Imaging Reporting and Data System, No. = number, US = ultrasound, Rad-score = radiomics score

## Discussion

In the current study, we retrospectively collected 312 BMUS and CEUS images of ACR TI-RADS 4 and 5 TNs, then developed and validated a dual-modal US radiomics nomogram that involving BMUS and CEUS Rad-scores, which outperformed the clinical combined US model and the dual-modal US Rad-score for the personalized prediction of benign and malignant TR4-5 TNs and meaningfully reduced the unnecessary FNAB rate compared with ACR TI-RADS. This easy-to-use graphical visualized tool might provide more accurate and robust information to promote clinical decision-making systems.

High-resolution ultrasonography is reckoned as the preferred diagnostic method for TNs. There have been several thyroid US risk stratification systems used in clinical practices, thereinto ACR TI-RADS was the most widely used stratification system due to its feasibility of classifying all the TNs [18]. However, there exists relatively low specificity and overlapping risk characteristics between benign and malignant suspicious TR4 and TR5 TNs [4]. In this study, the AUC, specificity, and unnecessary FNAB rate of the ACR TI-RADS stratification system for TR4-5 TNs were 0.653 (95% CI, 0.588–0.719), 42.4% (95% CI, 30.3-54.5%) and 32.0% in the training cohort and 0.669 (95% CI, 0.567–0.772), 44.4% (95% CI, 25.9-63.0%) and 36.9% in the validation cohort, respectively. Hence, to reduce the unnecessary FNAB rate and mitigate overdiagnosis and overtreatment, pursuing a non-invasive method with high specificity is necessary. In the past few decades, there have been numerous studies on the diagnosis of TNs using qualitative or quantitative CEUS [7, 19]. Even for differentiating benign and malignant TR4-5 TNs, using US facilitated by CEUS also has rather good performance, as CEUS provides effective supplementary micro- and macro-vascularization information within the TNs, which reflect the patterns of neoplastic growth [20, 21]. But radiologists’ subjective factors and inter-observer variability with different experiences in the visual interpretation of BMUS and CEUS videos could affect the diagnosis accuracy.

In recent years, “radiomics” as a machine-learning method has emerged in clinical practices to improve the accuracy of disease diagnosis, prediction, and prognosis as it can automatically extract high-throughput quantitative image features and detect information that is difficult to be assessed through visual interpretation. US-based radiomics methods have attracted the interest of numerous researchers for characterizing benign and malignant TNs using quantitative US image features [11–13]. Liang et al. reported that Rad-score composed of several dozen radiomics features extracted from grayscale US images outperformed the ACR TI-RADS evaluation of junior radiologists but reached no statistical difference with senior radiologists in predicting malignancy in TNs, which indicated the feasibility of radiomics method as a diagnostic tool [11]. Referring to differentiating benign and malignant TR4-5 TNs, Wang et al. developed an integrated system of combining deep learning network and traditional machine learning radiomics network to analyze suspicious solid or almost completely solid TNs. Although the performance of this integrated model was better than two senior and three junior ultrasonographers, it only got an AUC of 0.800 and an accuracy of 76.8% in the test set [4]. Wu et al. trained three deep learning convolutional neural networks and found that ResNet-50 performed the best and was superior to radiologists in discriminating benign and malignant TR4-5 TNs. But the performance of deep learning algorithms was weaker in the combined TR4 and TR5 datasets than separated TR4 dataset or TR5 dataset, with an AUC of 0.829 and accuracy of 78.4% in the independent external test set [14]. Our dual-modal US radiomics nomogram containing BMUS and CEUS images got an AUC of 0.873 and 0.851, and the accuracy of 84.0% and 80.7%, in the training and validation set, respectively, whose performance was superior to the clinical combined US model, dual-modal US Rad-score and the results of Liang et al. and Wu et al., indicating our developed dual-modal US radiomics nomogram was a valuable method to solve the actual difficulty of predicting benign and malignant TR4-5 TNs for radiologists in a real-world clinical diagnosis. A key factor contributing to the robustness of our dual-modal US radiomics nomogram could be the incorporation of BMUS and CEUS radiomics features, which differed from the research conducted by Liang et al. and Wu et al., which focused solely on grayscale US radiomics features for distinguishing between benign and malignant TR4-5 TNs.

CEUS radiomics analysis methods have been widely used in the field of disease diagnoses [22, 23], risk evaluation [24, 25], prognoses prediction [26, 27], and decision-making treatment [28]. To some extent, CEUS radiomics was more meaningful than BMUS radiomics as it could capture additional characteristics of blood flow information in addition to the extraction of grayscale US radiomics features [29]. However, most of the previous studies only applied grayscale US radiomics features in characterizing TNs and did not involve CEUS radiomics features. Our study result showed that the addition of BMUS radiomics features and CEUS radiomics features to the clinical combined US model notably increased the NRI and IDI, meaning that both BMUS Rad-score and CEUS Rad-score could be highly conducive markers for differentiating benign and malignant TR4-5 TNs. And CEUS Rad-score had noticeably higher NRI and IDI than the BMUS Rad-score, further demonstrating the considerable predictive value of CEUS imaging. This result was consistent with a previous study performed by Guo et al., which found that an AUC of 0.861 for the BMUS + CEUS radiomics model was superior to a single BMUS or CEUS Rad-score [30]. But their study had a rather small sample size of only 123 TR3-5 TNs in the entire dataset and 7 benign TNs in the validation dataset, which may cause overfitting and increased bias. Compared with their study, our developed dual-modal US radiomics nomogram further reduced the unnecessary FNAB rate considerably from 35.3 to 14.5% in the training cohort and from 41.5 to 17.7% in the validation cohorts in comparison to ACR TI-RADS.

As far as we know, our study represented the first attempt to evaluate the predictive value of a dual-modal US radiomics nomogram incorporating CEUS images in addressing the challenge of distinguishing benign and malignant TR4-5 TNs in an actual clinical setting. In the present study, we evaluated the clinical and US risk factors. Age, shape, margin, and enhancement direction were determined as significant predictive variables in a multivariate logistic regression analysis that were distinct from the BMUS and CEUS Rad-score. To facilitate decision-making, a dual-modal US radiomics nomogram was developed that integrated the six factors mentioned above, providing a user-friendly tool. This nomogram demonstrated excellent discrimination and calibration, surpassing the predictive efficacy of both the clinical and US risk factors prediction model and the dual-modal US Rad-score model in both the training and validation cohorts. The DCA further supported the effectiveness of the dual-modal US radiomics nomogram, indicating a significant improvement in predictive value for TR4-5 TNs compared with both the clinical combined US model and the dual-modal US Rad-score. To aid in the clinical utilization of the nomogram, we provided the sensitivity, specificity, positive predictive value, negative predictive value as well as accuracy for the model using the optimal cut-off value in evaluating the risk of TR4-5 TNs. When stratified into low- and high-risk subgroups based on the optimal cutoff value of the Nomo-score, we determined that TR4-5 TNs with a Nomo-score of 0.524 or higher represented a high-risk subset, with a high probability of malignancy (positive predictive value, 87.1%). Therefore, this high-risk subset may be candidates for further examination or treatment options.

Several limitations of our study should be considered. Firstly, this was a single-institution retrospective study that utilized a single vendor machine, which could result in selection bias and data imbalance and may not be applicable to other centers or machines. To validate the feasibility of our developed radiomics nomogram, a well-designed prospective longitudinal cohort study with a larger patient group and multi-vendor machines across multiple centers is essential in the future. Second, the CEUS Rad-score was only based on a single peak-enhancement CEUS image to represent the whole perfusion process, so some other information related to the dynamic CEUS videos that might be valuable to the TNs diagnoses might have been neglected. We anticipated further exploring more sophisticated and effective technical approaches to investigate the relationship between radiomics features and dynamic CEUS video characteristics (such as TIC parameters), which could potentially enhance the predictive performance of radiomics. Third, the scope of our study was solely restricted to TR4-5 TNs, and as such, our findings may not be applicable to TNs with lower TI-RADS scores.

## Conclusion

To sum up, this study developed a dual-modal US radiomics nomogram incorporating both BMUS and CEUS Rad-scores and clinical and US risk factors, demonstrating superior discrimination accuracy between benign and malignant ACR TI-RADS 4 and 5 TNs compared with the clinical combined US model and dual-modal US Rad-score and considerably reducing unnecessary FNAB rate in comparison to ACR TI-RADS. Moreover, it could guide further examination or treatment options.

### Electronic supplementary material

Below is the link to the electronic supplementary material.

Supplementary Material 1

## Data Availability

The datasets generated and/or analyzed during the current study are not publicly available due to privacy and ethical restrictions but are available from the corresponding author on reasonable request.

## Abbreviations

ACR: American College of Radiology
TI-RADS, TR: Thyroid Imaging Reporting and Data System
TNs: Thyroid nodules
US: Ultrasound
BMUS: B-mode ultrasound
CEUS: Contrast-enhanced ultrasound
FNAB: Fine needle aspiration biopsy
Rad-score: Radiomics score
AUC: Area under the receiver operating characteristic curve
ROI: Region of interest
ICC: Interclass correlation coefficient
LASSO: Least absolute shrinkage and selection operator
DCA: Decision curve analysis
IDI: Index integrated discrimination improvement
NRI: Net reclassification improvement
