# Supplementary material for: Dual-modal radiomics nomogram based on contrast-enhanced ultrasound to improve differential diagnostic accuracy and reduce unnecessary biopsy rate in ACR TI-RADS 4–5 thyroid nodules
Source: Cancer Imaging. 2024 Jan 23;24:17. doi: 10.1186/s40644-024-00661-3 (PMC10807093; doi:10.1186/s40644-024-00661-3)
Supplement: Supplementary file 1 — Supplementary Material 1 [file 40644_2024_661_MOESM1_ESM.docx]

Supplementary Table S1 Major packages of R software used in this study

| **Functions** | **R package** |
| --- | --- |
| Minimum redundancy maximum relevance (mRMR) algorithm | mRMRe |
| Least absolute shrinkage and selection operator (LASSO) regression | glmnet |
| Generate the receiver operating curve (ROC) and measure the area under the ROC (AUC) | pROC |
| ROC analysis to determine best cutoff value | OptimalCutpoints |
| Plot violin and bar diagrams | ggplot2 |
| Calibration curves | rms |
| Decision curve analysis (DCA) | rmda |


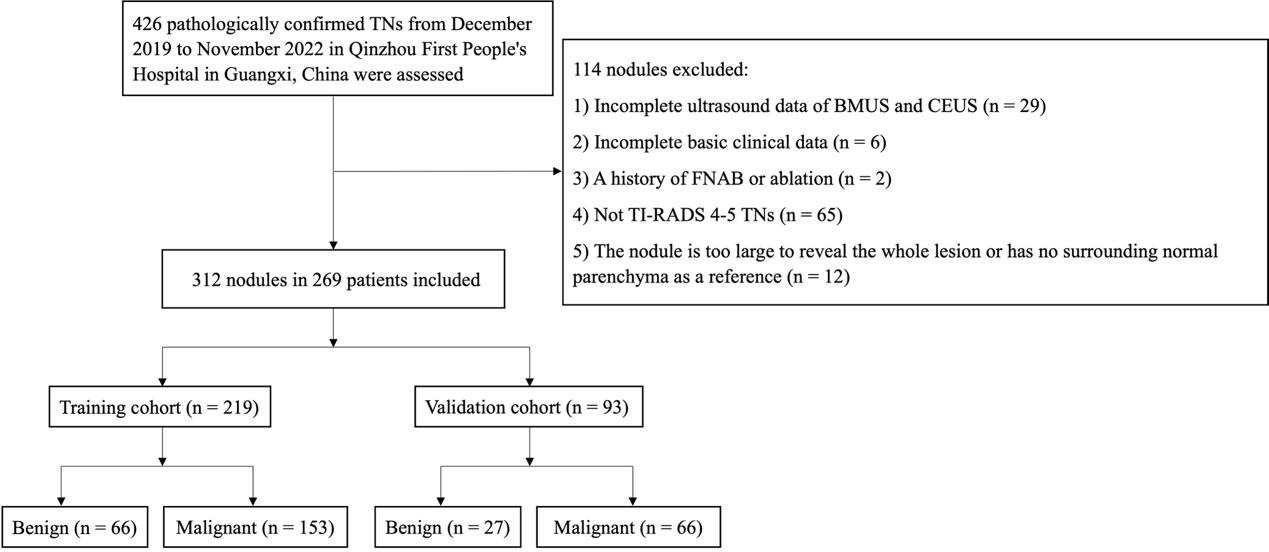


Supplementary Fig. S1

Flow diagram of the study population. TNs, thyroid nodules; BMUS, B-mode ultrasound; CEUS, contrast-enhanced ultrasound; FNAB, fine needle aspiration biopsy; TI-RADS, Thyroid Imaging Reporting and Data System.


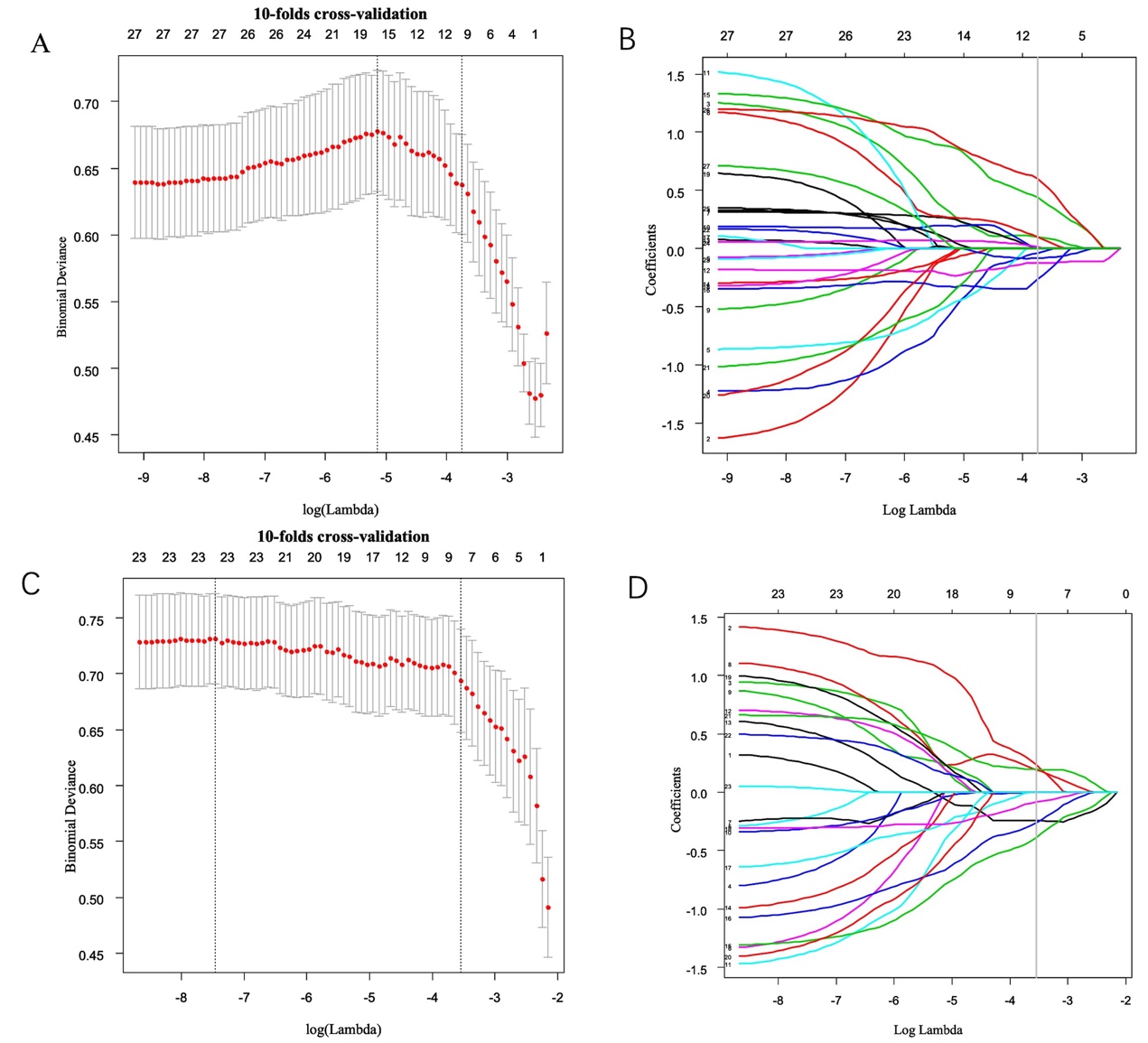


Supplementary Fig. S2

TI-RADS 4-5 TNs-related feature selection using the LASSO logistic regression algorithm in the training cohort. (A, C) The ten-fold cross-validation and the minimal criteria process were used to generate the optimal penalization coefficient lambda (λ) of 0.02363 and 0.02879 for the BMUS (A) and CEUS (C) features in the LASSO model, respectively. LASSO coefficient profiles of the BMUS (B) and CEUS (D) features. The dotted vertical line was drawn at the value selected by ten-fold cross-validation when the optimal λ resulted in ten (BMUS) and seven (CEUS) nonzero coefficients, respectively. TI-RADS, Thyroid Imaging Reporting and Data System; LASSO, least absolute shrinkage and selection operator; BMUS, B-mode ultrasound; CEUS, contrast-enhanced ultrasound.

Supplementary A1 Detailed time-intensity curve (TIC) analysis procedures of contrast-enhanced ultrasound (CEUS) videos.

VueBox® is an external offline dynamic CEUS video analysis software with the ability to generate the quantitative perfusion TIC parameters. One radiologist with 10 years of experience in thyroid ultrasound, who was blinded to all the clinicopathological information of TNs, performed the VueBox® software analysis. First, the dynamic CEUS videos in DICOM format were input into VueBox® software. Second, select the area of interest (ROI): 1) boundary ROI: outline the thyroid area to be analyzed, including thyroid nodules and as large a range of peripheral thyroid tissue as possible; (2) lesion ROI: outline the entire thyroid nodule to be analyzed; (3) reference ROI: draw a region of peripheral thyroid tissue within the boundary ROI, and try to select thyroid tissue at the same depth as the lesion ROI. If necessary, motion correction (which compensates for respiratory motion) was used, which can reflect the results more accurately.

A TIC was analyzed using the following parameters: (1) peak intensity (PI), defined as the maximal signal intensity measured in the lesion ROI and reference ROI; (2) time to peak (TTP, in seconds), defined as the time from the starting point to the PI of the curve.

Supplementary A2 The BMUS and CEUS radiomics score formulas were constructed. Rad-score = intercept + coefficient × radiomics features, and the radiomics score of each nodule was calculated.

BMUS Rad-score = 0.896

- 0.266*square_ngtdm_Strength

- 0.123*wavelet.L_ngtdm_Busyness

- 0.082*logarithm glszm LargeAreaLowGrayLevelEmphasis

- 0.037*original gldm DependenceNonUniformityNormalized

+ 0.002*wavelet.H firstorder Mean

+ 0.013*exponential glcm MCC

+ 0.099*wavelet.H glszm SmallAreaHighGrayLevelEmphasis

+ 0.101*original glszm SmallAreaLowGrayLevelEmphasis

+ 0.438*square gldm SmallDependenceLowGrayLevelEmphasis

+ 0.601*exponential gldm DependenceEntropy

CEUS Rad-score = 0.877

- 0.388*squareroot glszm LargeAreaLowGrayLevelEmphasis

- 0.263*squareroot ngtdm Busyness

- 0.244*original firstorder Kurtosis

- 0.086*logarithm glszm LargeAreaLowGrayLevelEmphasis

+ 0.194*wavelet.H gldm DependenceEntropy

+ 0.195*exponential glcm Idmn

+ 0.234*original firstorder Skewness
